# Superspreading events in the transmission dynamics of SARS-CoV-2: Opportunities for interventions and control

**DOI:** 10.1371/journal.pbio.3000897

**Published:** 2020-11-12

**Authors:** Benjamin M. Althouse, Edward A. Wenger, Joel C. Miller, Samuel V. Scarpino, Antoine Allard, Laurent Hébert-Dufresne, Hao Hu

**Affiliations:** 1 Institute for Disease Modeling, Bellevue, Washington, United States of America; 2 University of Washington, Seattle, Washington, United States of America; 3 New Mexico State University, Las Cruces, New Mexico, United States of America; 4 School of Engineering and Mathematical Sciences, La Trobe University, Bundoora, Victoria, Australia; 5 Network Science Institute, Northeastern University, Boston, Massachusetts, United States of America; 6 Department of Marine & Environmental Sciences, Northeastern University, Boston, Massachusetts, United States of America; 7 Department of Physics, Northeastern University, Boston, Massachusetts, United States of America; 8 Department of Health Sciences, Northeastern University, Boston, Massachusetts, United States of America; 9 ISI Foundation, Turin, Italy; 10 Santa Fe Institute, Santa Fe, New Mexico, United States of America; 11 Département de Physique, de Génie Physique et d’Optique, Université Laval, Québec, Québec, Canada; 12 Centre Interdisciplinaire en Modélisation Mathématique, Université Laval, Québec, Québec, Canada; 13 Vermont Complex Systems Center, University of Vermont, Burlington, Vermont, United States of America; 14 Department of Computer Science, University of Vermont, Burlington, Vermont, United States of America; 15 Bill & Melinda Gates Foundation, Seattle, Washington, United States of America

## Abstract

Severe Acute Respiratory Syndrome Coronavirus 2 (SARS-CoV-2), the etiological agent of the Coronavirus Disease 2019 (COVID-19) disease, has moved rapidly around the globe, infecting millions and killing hundreds of thousands. The basic reproduction number, which has been widely used—appropriately and less appropriately—to characterize the transmissibility of the virus, hides the fact that transmission is stochastic, often dominated by a small number of individuals, and heavily influenced by superspreading events (SSEs). The distinct transmission features of SARS-CoV-2, e.g., high stochasticity under low prevalence (as compared to other pathogens, such as influenza), and the central role played by SSEs on transmission dynamics cannot be overlooked. Many explosive SSEs have occurred in indoor settings, stoking the pandemic and shaping its spread, such as long-term care facilities, prisons, meat-packing plants, produce processing facilities, fish factories, cruise ships, family gatherings, parties, and nightclubs. These SSEs demonstrate the urgent need to understand routes of transmission, while posing an opportunity to effectively contain outbreaks with targeted interventions to eliminate SSEs. Here, we describe the different types of SSEs, how they influence transmission, empirical evidence for their role in the COVID-19 pandemic, and give recommendations for control of SARS-CoV-2.

## Abstract 2

This Essay discusses how the transmission of COVID-19 is dominated by superspreading events, proposing that finding ways to cut the long tail of secondary infections is important for controlling onward transmission.

## Introduction

While Severe Acute Respiratory Syndrome Coronavirus 2 (SARS-CoV-2) has moved swiftly around the globe, causing millions of Coronavirus Disease 2019 (COVID-19) cases, much attention has been given to the basic reproduction number (R_0_), estimated to be roughly between 1.5 and 4 [1]. As the virus spread, it has become clear that relying on a single value to characterize the number of secondary infections—and thus estimates of the transmissibility of this virus—is inadequate to capture the true transmission dynamics and subsequent risk to humanity [2]. Indeed, a litany of official reports and anecdotes have identified key superspreading events (SSEs), which have propelled transmission and infected many. We consider SSEs to be a single generation of spread where an individual gives rise to a large number of secondary infections.

Earliest notable examples include a Briton who returned from a Singapore conference and infected 13 other people in a ski resort in the Alps [3], more than 70 cases were linked to a Boston Biogen conference within 2 weeks [4], and the most extreme example so far is South Korea “Patient 31” who caused an SSE that eventually resulted in a cluster of more than 5,000 cases in Daegu [5]. A Hong Kong resident visited the Diamond Princess cruise ship on January 25, 2020 and later tested positive. The number of positive cases on the cruise quickly rose to about 700 people or 17% of all passengers within 20 days [6]. In Chicago, 15 cases stemmed from 1 person at multiple family gatherings [7], and there are multiple reported clusters of 2 to 48 cases after having had meals together [8].

In March 2020, multiple European countries simultaneously reported unusually large numbers of imported cases from Ischgl, Austria, a popular ski town. Epidemiological investigations found that infections might have been circulating since late February 2020, and the individual causing the SSEs might have been a bartender working in an après ski bar who subsequently was diagnosed positive [9]. In New York, a lawyer was infected and spread it to at least 50 others in New Rochelle [10]. In Mount Vernon, Washington, after a choir rehearsal on March 10, 2020, 45 out of 60 choir members fell ill, and 28 of those 45 tested positive for SARS-CoV-2. No one appeared to be sick during the rehearsal [11]. Investigations revealed that the index patient in this case directly infected 52 others [12]. An Indian preacher died after returning from a trip to Italy and Germany and attending a large gathering to celebrate the Sikh festival of Hola Mohalla. A week later, at least 19 of his relatives were infected, and it resulted in a quarantine of 40,000 residents in Punjab [13].

In April and May 2020, universal testing at a Boston homeless shelter found that 36% of residents were tested PCR–positive [14], and an 87-year-old man in Harbin, China directly infected more than 78 individuals within a few days at home and 2 hospitals [15]. Singapore has seen a sharp rise in cases, with the vast majority (88%) being linked to dormitories. S11, a 10,000-person capacity dormitory, has the largest cluster in Singapore, with more than 2,200 infections [16]. More than 100 cases were traced back to nightclubs in Seoul that were visited by a young man who later tested positive [17]. In Chennai, India, the Koyambedu vegetable market has emerged as a hotspot, with more than 2,000 cases traced to the market [18]. While this example and that of the Singaporean dormitory are clusters of cases and not single SSEs, they are composed of multiple SSEs happening in succession.

These accounts in addition to the many examples of SSEs in long-term care facilities [19], prisons [20,21], meat processing facilities [22], and fish factories [23] demonstrate the central role played by SSEs on the transmission dynamics—and subsequent epidemiological control of—SARS-CoV-2. Here, we describe the potential types of SSEs, how they influence transmission, and give recommendations for control of SARS-CoV-2.

## Superspreading events, stochasticity, and negative binomially distributed secondary infections

**Fig 1:**
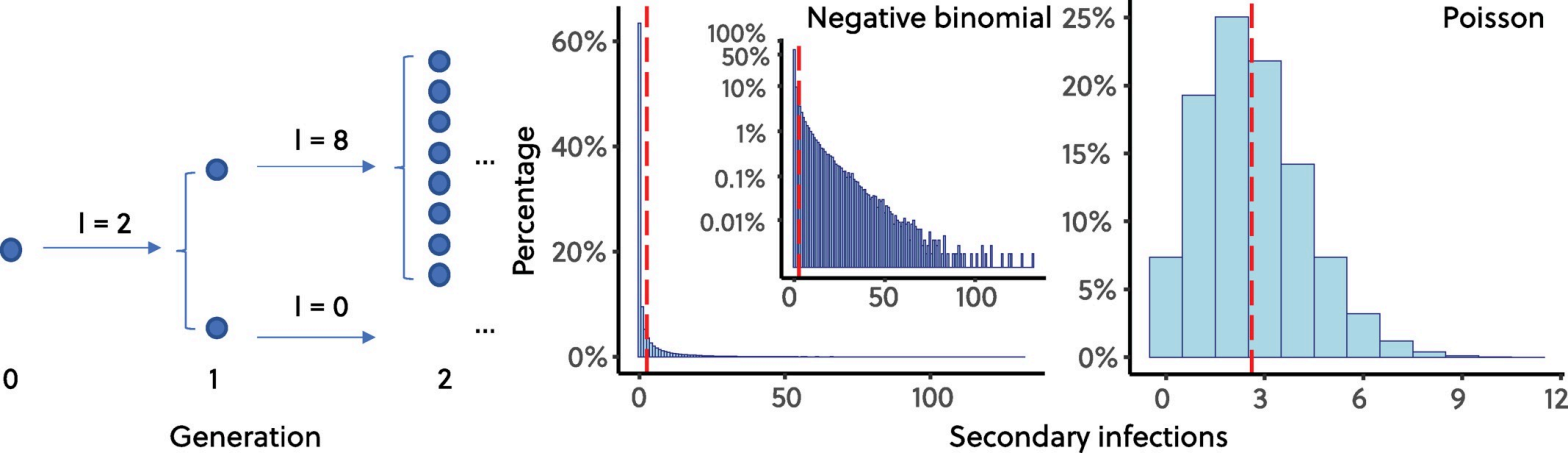
Poisson and NB-distributed secondary infections. Figure shows a schematic of an NB branching process and example distributions of NB and Poisson distributions of secondary infections. Both distributions have **R**_**0**_ = 2.6 [28], and the NB has dispersion parameter ***k*** = 0.16 [25] with 100,000 draws. The code required to generate this figure can be found in S1 Data. NB, negative binomial.

Many classical models in epidemiology either assume or result in a Poisson distribution of secondary infections per infected individual. Because Poisson distributions have the same mean and variance, they often fail to capture the relevant features of SSEs. As a result, SSEs are now commonly modeled using a negative binomial (NB) distribution of secondary infections per infected individual [24]. NB distributions can be parameterized with a mean (thought of as R_0_) and a dispersion parameter, *k*, where smaller values of *k* indicate a longer tail (over-dispersion). When *k* is close to 0, even with a high R_0_, most individuals give rise to 0 or 1 secondary infections, and few give rise to many infections—a so-called “long tail” of infections. However, as *k* grows larger, an NB distribution approaches a Poisson distribution (becoming exactly Poisson when *k*→∞), and the effect of SSEs on the epidemic decreases. In Fig 1, we show a schematic of an NB branching process and distribution of NB and Poisson distributions of secondary infections under the same R_0_. Earlier transmission modeling studies suggest that the offspring distribution of SARS-CoV-2 is highly over-dispersed with *k* comparable to that of Severe Acute Respiratory Syndrome Coronavirus 1 (SARS-CoV-1) [25]. Recent studies in Israel [26] and Hong Kong [27] have found that 10% to 20% cases are responsible for 80% of local transmission, consistent with earlier findings. We make note that—even for the same pathogen—SSEs have many potential causes, both biological and social (which we outline below), while in conceptualizing and modeling such SSEs, we use NB distributions, which are parameterized by 2 variables, R_0_ and *k*, which encompass the myriad processes that give rise to SSEs.

**Fig 2:**
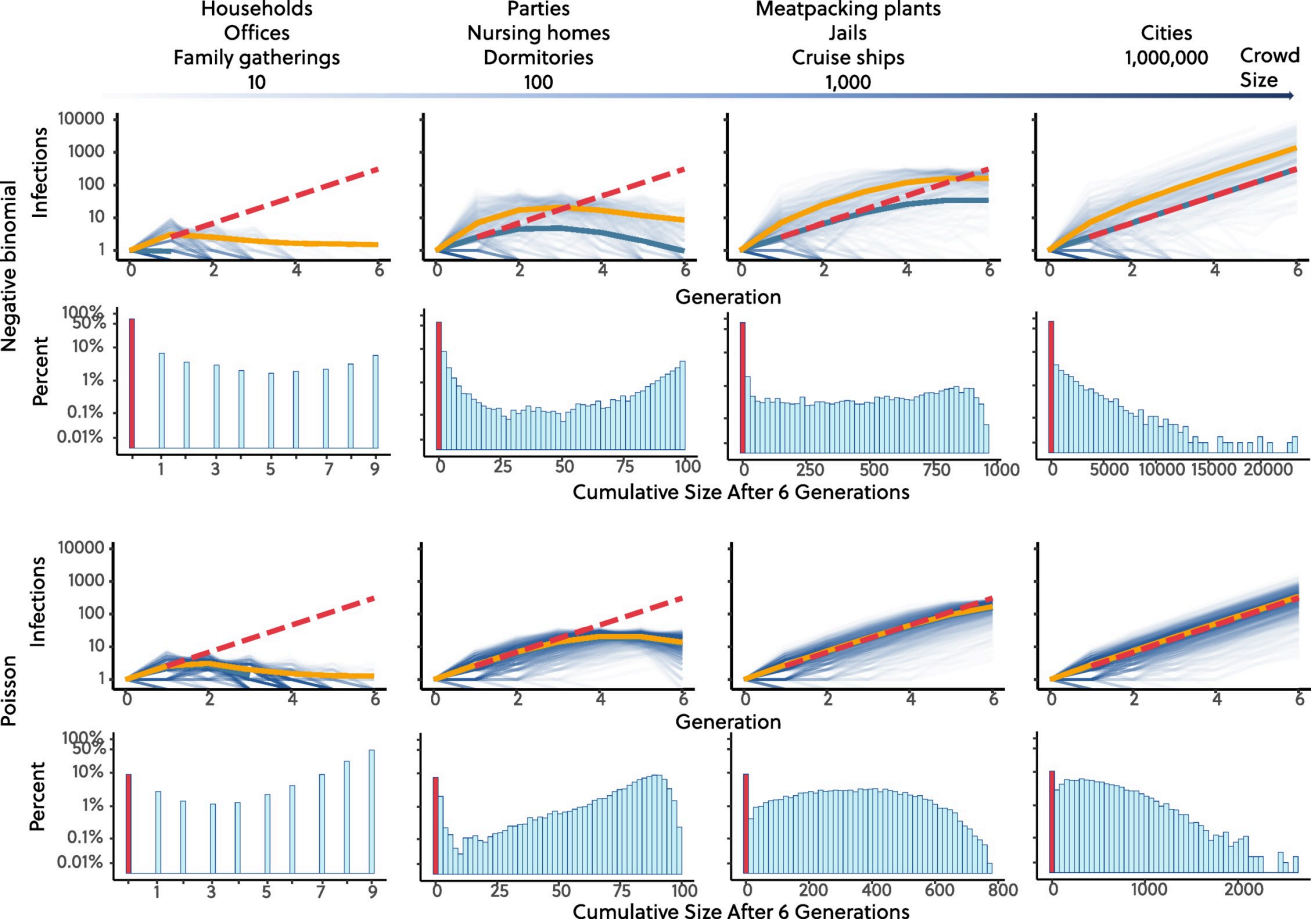
Example trajectories of NB and Poisson branching processes. Figure shows example trajectories (in number of active infections versus generation) of NB and Poisson branching processes and cumulative infection sizes after 6 generations of spread. Both simulations start with 1 infection and have the same **R**_**0**_ = 2.6. For NB branching process, we assume dispersion parameter *k* = 0.16, same as SARS-CoV-1 [24,33]. We run all simulations 10,000 times. Dashed red lines represent theoretical values in the large-population limit $I=R_{0}^{n}$, where *I* is number of active infections, and *n* is number of generations. Solid blue lines are the mean values of all simulations including those that have not taken off, which overlap with the theoretical values when the susceptibles are not depleted. Solid orange lines are the mean value for simulations that took off, and the outbreaks appear more explosive in the first few generations in the NB simulations. Both number of active cases and cumulative infections are in **log**_**10**_ scale. The code required to generate this figure can be found in S1 Data. NB, negative binomial; SARS-CoV-1, Severe Acute Respiratory Syndrome Coronavirus 1.

An outbreak dominated by SSEs and an NB distribution of secondary infections with small *k* has distinct transmission features as compared to a Poisson distributed, “mean-field” outbreak with the same R_0_. First, secondary infections will be over-dispersed, such that early dynamics are more stochastic, and an outbreak is much less likely to grow into a large epidemic. In Fig 2, we show an example with both Poisson and SARS-CoV-1-like NB distributions (*k* = 0.16) under the same mean R_0_ = 2.6 [28] and with different population sizes ranging from small clusters of 10 (like households) to large ones of 10^6^ (like cities). To incorporate randomness, we utilize stochastic branching process versions of each model. To illustrate the vast difference between these models, we plot the transmission dynamics across 6 generations or 24 to 36 days assuming the 4- to 6-day serial interval of COVID-19 [29–32].

Comparing the 2 models, we show that for the NB model—in all cases where the population size was at least 100—there were no secondary infections in 63% of outbreaks, and in 77% of outbreaks, there were both less than 10 total infections, and ongoing transmission failed to establish, compared with 7% with no secondary infections and 11% with less than 10 total infections for the Poisson model. This vast difference, where outbreaks fizzle out under the NB, but grow large under the Poisson model, is due to the over-dispersion of the SARS-CoV-1-like NB distribution: As the probability of giving rise to 0 or 1 additional case increases, onward transmission becomes less likely to establish. However, if the outbreak takes off under the NB model—and increases beyond 1 or 2 SSEs, i.e., often only a few dozen cases—the dynamics begin to show stable exponential growth, with a growth rate approaching that of a model with the same R_0_, but a Poisson distribution of secondary infections per infected individual, i.e., an NB with *k* much greater than 0. Nevertheless, during the early phase of an NB outbreak that takes off, the epidemiological curve, i.e., new cases over time, will appear more explosive (represented in estimated R_0_ based on case counts) in the first few generations when SSEs will generate the vast majority of secondary infections, making it possible to spin out large infection clusters in few numbers of generations, whereas a Poisson model cannot. An example is shown in Fig 2 where the outbreak appears more explosive (orange line versus blue line) if we only consider outbreaks that took off and also consider the outbreak aboard the Diamond Princess cruise where 135 cases were seen within 5 days [6]. Lastly, and importantly, to establish and stabilize exponential growth of an outbreak under the NB model, a continuous fuel of SSEs is necessary and an outbreak can be brought largely under control—the effective reproduction number R_eff_ is significantly reduced—when SSEs, which drive much of the transmission, are eliminated. As we discuss below, our ability to intervene and eliminate/reduce SSEs depends heavily on the underlying sociobiological mechanisms causing them (see Box 1).

**Box 1. Characterizing superspreading events**

The total number of secondary infections from an infected individual is based on 2 variables: number of susceptible contacts (potential infectees) and probability of infection per contact. SSEs can occur from either an infectious individual having close contacts with many susceptible individuals and/or having a higher probability of transmission per contact, or it can be an opportunistic phenomena where number of contacts and/or probability of contact is high in an unusual way, like festivals, bars, or social gatherings. There are at least 4 types of SSEs which we identify below. We note that these categories are neither exhaustive nor exclusive: There may be situations that could be placed in multiple categories, but these categories capture the majority of SSE types (see also the Supporting information of Lloyd-Smith and colleagues [24] for additional discussion of SSE types).

- Biological. Individuals with a higher probability of transmitting per contact. These may be hard to identify a priori; for infections with most pathogens, pathogen loads may vary over many orders of magnitude across individuals [34]. For SARS-CoV-2, individual-level viral loads are dependent on time since onset and might be associated with demographics like age and disease severity [35]. The temporal profile of SARS-CoV-2 viral load peaks at or just before the onset of symptoms and decreases quickly to near the PCR detection threshold within a week [31,35,36]. Large heterogeneities in viral load—up to 8 log_10_ differences—exist between individuals [37].
- Behavioral/social. Individuals causing SSEs may have a higher number of susceptible contacts per person. Numerous studies have demonstrated marked differences in individual contacts by profession and over time.
- High-risk facilities and places such as meat-packing plants, workers’ dormitories, prisons, long-term care facilities, or healthcare settings. The nature of interactions in these places seem to repeatedly place individuals at higher risk of acquiring and transmitting infection. Importantly, this driver of SSEs can lead to spillover into the larger community. Controlling a broader outbreak will be very difficult if there is a focal hotspot, which is continually seeding new transmission chains, as seen in other respiratory pathogens such as tuberculosis [38,39].
- “Opportunistic” scenarios. The first scenario is when larger numbers of individuals temporarily cluster, and even with an average probability of transmission per contact, people are briefly far above their “average” number of susceptible contacts. The second scenario is that the probability of transmission per contact is temporarily increased in an unusual way, such as singing or frequent loud speaking. These 2 opportunistic scenarios are more frequently seen in outbreaks at nightclubs, cruise ships, crowded public transportation, parties, choirs, or other mass gathering events. Think of an individual infected with SARS-CoV-2 who walks into a room with 100 others who are all completely susceptible to infection. That person has the opportunity to infect all of them, causing an SSE. Now, take that same individual, and put them in a room where all 100 others are completely immune to infection. The infectious individual can infect 0 of the people in the room, and there is no SSE. The same individual placed in 2 different situations can or cannot initiate an SSE based on the situation.

In those cases where an individual’s connectivity plays a role, it is generally expected that these individuals will also be disproportionately likely to become infected as well [40,41]. These may be easier to identify in advance, but the other cases may be more difficult to prevent.

## Where do superspreading events take place?

Since the beginning of the outbreak, many types of SSEs have been reported with increasing frequency. Although the data are still scarce, some patterns have started to emerge on where and under what circumstances SSEs occur: Closed environments [42], environments with poor ventilation, crowded places, and long durations of potential exposure all correlate with emergence of SSEs. These SSE “hotspots” are both a source of infection for the community as well as potential targets for intervention. Hotspots are emerging as seeding infection in small metro areas as large outbreaks have occurred in meat-packing plants and prisons. Many clusters were linked to a wide range of mostly indoor settings, like households, public transport, hospitals, parties, bars, elderly care centers, and schools [43,44].

The stochastic characteristics of early transmission resulting in a dichotomy of frequent extinctions with rare but explosive outbreaks are reflected in a few examples. As of May 16, 2020 in Ohio, only 3 out of 16 jails with positive cases had large outbreaks of attack rate larger than 10% [45]. In King County, Washington, a total of 105 long-term care facilities reported positive cases, but only 19 of them had 5 or more deaths indicating potentially large outbreaks within facilities [46]. Among a total of 47 cruise ships reporting positive cases or COVID-19-related deaths, only a few had notable large outbreaks, i.e., Diamond Princess, Ruby Princess, Grand Princess, Celebrity Apex, Horizon, Greg Mortimer, and Costa Atlantica [47].

In January and February 2020, initial clusters of explosive outbreaks did not take off first at the expected dense metropolitan areas and air transit hubs (such as Seoul, Tehran, Paris, London, or Frankfurt), but instead started in neighboring small cities: Daegu in South Korea, Gangelt in Germany, Qom in Iran, and Lombardy in Italy. This can partially be explained by considering that outbreaks are much more likely to occur when seeded by an SSE, and while large population centers will have more introductions than any given smaller city, there are many small cities, and the initial large outbreaks occur in whichever locations the first SSEs occur. Therefore, incidence data can look more random and relevantly unpredictable than expected if we only use population density and air traffic data as sole predictors. This phenomenon could be thoroughly explored using an individual- or agent-based simulation that explicitly uses air and ground transportation data [48,49].

Because they play an important role in the spread of infection, hotspots pose an opportunity for surveillance and control: Focusing on facilities and activities known to sustain hotspots, such as healthcare facilities, nursing homes, prisons, meat-packing plants, homeless shelters, schools, and mass gatherings, as well as those places with closed, poorly circulated environments, can provide efficient ways to identify potential SSEs before they happen, therefore potentially reducing a substantial amount of transmission in the population.

## Controlling outbreaks with targeted interventions: “Cutting the tail”

**Fig 3:**
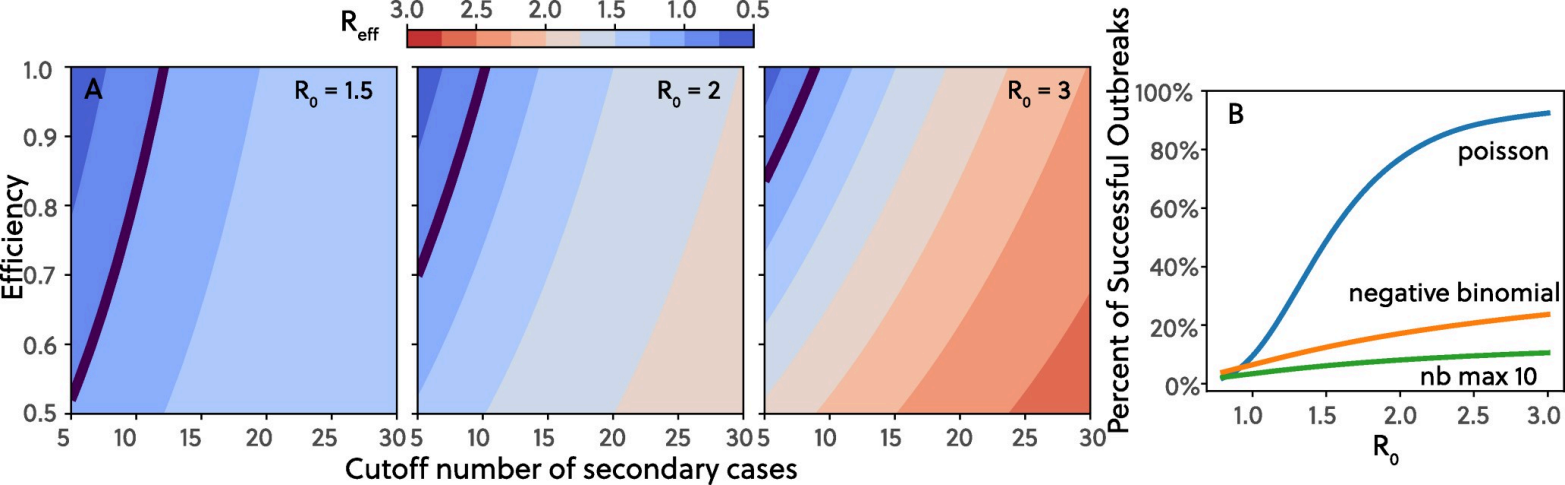
Controlling outbreaks and the effect of “cutting the tail”. (A) Values of **R**_**eff**_ under different thresholds of maximum number of secondary infections and the probability of keeping the maximum number of secondary infection thresholds. We compare 3 scenarios with **R**_**0**_ = 1.5, 2.0, and 3.0, where lower **R**_**0**_ represents the consequence of population-wide mild to moderate NPIs. The NB distribution is truncated at the max number of secondary cases, and the distribution is renormalized after the truncation. Contours highlight **R**_**eff**_ = 1 boundary. Lower **R**_**0**_ facilitates the extinction of outbreak, and increases the probability of success controlling the outbreak, especially when the probability of reducing hotspot transmission is low. If the vast majority of transmission inside hotspots can be eliminated under all **R**_**0**_ scenarios, a control target of less than 10 secondary infections can bring **R**_**eff**_ to close to unity. (B) Probability of an outbreak successfully taking off with 1 seed infection under NB and Poisson distributions, as well as an NB distribution with cutoff number of secondary infections of 10 and with 100% efficiency. The stochastic branching process simulations are run 10,000 times with **R**_**0**_ ranging from 0.8 to 3.0. The success of outbreak is defined as having more than 20 infections at generation 6. Dispersion parameter *k* for NB is 0.16. Data are calculated using methods based on probability-generating functions [50]. The code required to generate this figure can be found in S2 Data. NB, negative binomial; NPI, non-pharmaceutical intervention.

Although they are rare, SSEs seem to play a major role in determining the growth trajectory of an epidemic. Thus, “trimming” the long tail of large secondary infections is important for outbreak control and suppression (whether through targeted interventions [24] or broad social distancing). To demonstrate how preventing SSEs can efficiently decrease R_eff_, we first assume that controlling SSEs are the only mechanism used to lower R_eff_ and that SSEs above a certain size are targeted for control. For now, we avoid describing how these events are controlled and instead simply demonstrate the importance of doing so. In the contour plots in Fig 3A, we show how R_eff_ decreases when SSEs above a specified threshold size, *N*_cutoff_, instead infect a number of individuals smaller than *N*_cutoff_. The fraction of SSEs where the intervention was successful is controlled by an efficiency parameter (from 0.5 to 1), where 0.5 indicates that 50% of SSEs above the threshold size, *N*_cutoff_, are controlled. Using this intervention model and assuming R_0_ = 3, we find that by only targeting a small number of individuals with more than 10 secondary infections (about 10% of all secondary infections) with 50% to 100% efficiency, the mean R_eff_ is reduced from 3 to 2.06 and 1.09, respectively.

Next, we consider a scenario where additional non-pharmaceutical interventions (NPIs) or behavioral changes, referred to collectively as NPIs, bring the population-wide R_eff_ down to 2 or 1.5, prior to efforts targeted specifically at SSEs. If the NPIs bring R_eff_ down to 1.5, then using an SSE intervention threshold of 10 secondary infections will reduce R_eff_ to 0.85 or 1.21 under 100% and 50% efficiency, respectively. What becomes clear is that when transmission is dominated by SSEs, “cutting the tail” interventions are more effective and impactful than reducing population-wide R_eff_. These results suggest that policymakers should focus on preventing transmission events where over 10 people are infected using industry-specific mitigation efforts. This effect—that targeting SSEs is more effective at reducing R_eff_, can also be seen in Fig 3B, where the probability of a large outbreak—defined as having more than 20 cumulative infections by generation 6—decreases as SSEs are controlled. More specifically, holding R_0_ constant and “cutting the tail” of SSEs, the probability of starting large outbreaks drops rapidly. By intervening to reduce the size and frequency of SSEs, transmission chains “collapse” more frequently, because there are not enough SSEs to fuel their growth, and outbreaks fizzle before exploding into widespread epidemics.

Efforts should be spent to understand how to reduce transmission in the 4 types of SSEs. For the biological type, it is important to understand the characteristics of individuals causing SSEs as interventions targeted at these individuals have the potential to be highly efficient and effective [24]. For example, what individual-level attributes and behaviors lead to SSEs, and how to identify them quickly? As viral loads decrease over time and most transmissions are front-loaded, timely identification and/or isolation becomes crucial. If future studies reveal meaningful viral load differences by demographics—for example, a difference by age or sex—and that there exists meaningful differences in transmission across viral loads, a transmission risk classification algorithm, similar to the algorithm stratifying high risk of hospitalization and death [51,52], can be used together with individual contact networks to stratify individuals, and remind them the potential of causing SSEs as they are reported sick and need to be tested. PCR test results could potentially include a rough category of transmissibility by cycle threshold values, for example, reporting a high transmission risk for those with cycle thresholds less than 20. This might help people avoid crowded events and reduce their contacts. Furthermore, rapid, highly specific but modestly sensitive test should be considered to eliminate superspreading hotspots. Such tests might not find all infections, but given that most infections don’t have onward transmission, if the test is very effective in identifying high transmitters, it has great potential to interrupt a substantial portion of transmission by testing early and frequently [53]. Environmental surveillance might play an important role in identifying the hotspots if viral load in stools from individuals causing SSEs are also orders of magnitudes greater [54,55].

For the behavioral/social type, a complete contact network for an individual can be estimated using surveys or cell phone tracking and used to identify 2 kinds of people—those with a high degree, meaning lots of close contact per day and those with high centrality, meaning they are crucial in connecting different population clusters. While this would be logistically difficult and poses ethical issues, even partial reconstruction of an individual’s contact distribution would be tremendously useful for identifying individuals with high numbers of potential infection events. Personalized messages or tests could be targeted at these individuals, stressing the need for better infection control.

For both high-risk facilities and “opportunistic” scenarios, to effectively prevent transmission in hotspots, it is important to understand the indoor transmission routes of SARS-CoV-2, which might include droplets and aerosols through coughing, sneezing, and speaking [56–58], fecal–oral [59–61], or indirectly through surfaces [62]. A recent study estimates that 1 minute of loud speaking generates at least 1,000 virion-containing droplet nuclei and could remain airborne for more than 8 minutes [58], suggesting a possible mechanism of creating large exposure and starting SSEs. Contact tracing is a useful tool to reveal the relative importance of different transmission routes in a variety of hotspots, for example, in restaurants [63], call centers [64], choirs [12], and churches [65]. More of these real-world examples will allow policymakers to evaluate the effectiveness of different control measures—for example, reducing crowd density, fever screening, rearranging seating layout, requiring masks, and good ventilation—in eliminating major transmission routes in confined spaces. Finally, directing interventions toward the most vulnerable populations (e.g., nursing homes and other long-term care facilities) would efficiently protect those at greater risk for COVID-19-related morbidity and mortality.

## Stochasticity, superspreading events, and the future of SARS-CoV-2

Multiple lines of evidence at the individual and population level strongly indicate the role of SSEs in the transmission dynamics of SARS-CoV-2 and that we should not overlook the heterogeneity in numbers of secondary infections [66]. Our mental picture should not be that most people transmit to 2 or 3 other people, but instead a small number of infections dominate the transmission while most others fail to have secondary infections. The distribution of R_0_ is over-dispersed with a high probability of extinction on the lower end and a long tail on the higher end.

Outbreaks will be less likely to take off because of the high probability of extinction. At the early stage, we will see more randomness and stochasticity, and it seems more explosive with huge number of cases reported in the first few generations. But once it takes off, it still becomes a stable exponential as per classic deterministic models. Behavioral change, mild to moderate NPIs to lower population-wide R_eff_, as well as other factors such as population density [67] and climate [68], might be more impactful, as a lower population R_eff_ increases the extinction probability and increases the success probability of “cutting the tail.” Therefore, all measures that could potentially reduce R_0_ should be considered and implemented, even if some only have a minor impact.

Because SSEs are fueling this outbreak, we have an opportunity to take advantage of this heterogeneity in transmission and use it to risk-stratify populations and locations for public health interventions and interrupt future SSEs. Many of recent outbreaks such as in Florida, Texas, California, the Montréal metropolitan area, and in Spain (Catalunya and Aragón) were either linked to the reopening of indoor dining, bars, nightclubs, or private social gatherings where physical distancing wasn’t respected, or linked to workers living in close quarters. Therefore, even though some governments took drastic measures to limit the spread of SARS-CoV-2, the loosening of these measures has blind spots, which may not be very large, but are large enough for SSEs to occur.

Novel methods are needed to quickly predict, identify, or isolate individuals/hotspots with the potential for causing SSEs. It may prove difficult to identify and isolate individuals with the potential for causing SSEs and infectious individuals that still transmit. This is compounded with the sizeable proportion of presymptomatic as well as asymptomatic or minimally symptomatic individuals who can actively transmit [69–72]. If SSE predictors cannot be identified, then every infectious individual has the opportunity to cause an SSE if exposed to sizable susceptible populations. Therefore, it is crucial to understand types of hotspots and patterns of transmission for each type, as interventions might have to focus on all hotspots at high risk for SSEs and limiting gatherings at these places and/or through rapid and extensive testing and contact tracing (both traditional and digital) to identify presymptomatic and asymptomatic people [73,74]. Several countries, such as South Korea [75], Singapore [76], and Iceland [77], are great examples showing that it is possible to contain the outbreak through extensive testing, identification of hotspots, contact tracing, and quarantine, without relying on extensive lockdowns. Contact tracing efforts should have an explicit goal to understand types of transmission and hotspots, so that the characterization of transmission could be used to adapt and prioritize other recommendations such as masks and mass gatherings. Such contact tracing data could also be used to reconstruct individual contact patterns useful to identify high-transmission-risk individuals. Such data should be made available to researchers to facilitate identification of high-risk groups. Further, it thus remains important to be cautious in reopening populations undergoing cordons sanitaires until transmission routes in different types of hotspots are well understood or when safe and effective COVID-19 treatments and vaccines are available.

## Supporting information

S1 Data This contains Python code to generate Figs 1 and 2. (ZIP)

S2 Data This contains Python code to generate Fig 3. (ZIP)

## Abbreviations

COVID-19: Coronavirus Disease 2019
NB: negative binomial
NPI: non-pharmaceutical intervention
SARS-CoV-1: Severe Acute Respiratory Syndrome Coronavirus 1
SARS-CoV-2: Severe Acute Respiratory Syndrome Coronavirus 2
SSE: superspreading event
